# TRiC/CCT chaperonin is required for the folding and inhibitory effect of WDTC1 on adipogenesis

**DOI:** 10.3389/fcell.2023.1225628

**Published:** 2023-08-24

**Authors:** Wen-Shuai Tang, Xiang Cen, Shan-Shan Yao, Shu-Ting Yin, Li Weng, Tong-Jin Zhao, Xu Wang

**Affiliations:** ^1^ State Key Laboratory of Cellular Stress Biology, School of Life Sciences, Xiamen University, Xiamen, China; ^2^ State Key Laboratory of Genetic Engineering, Shanghai Key Laboratory of Metabolic Remodeling and Health, Institute of Metabolism and Integrative Biology, Fudan University, Shanghai, China; ^3^ School of Life Science, Anhui Medical University, Hefei, Anhui, China

**Keywords:** TRiC/CCT, WDTC1, adipogenesis, protein folding, obesity

## Abstract

Obesity has become a global pandemic. WDTC1 is a WD40-containing protein that functions as an anti-obesity factor. WDTC1 inhibits adipogenesis by working as an adaptor of the CUL4-DDB1 E3 ligase complex. It remains unclear about how WDTC1 is regulated. Here, we show that the TRiC/CCT functions as a chaperone to facilitate the protein folding of WDTC1 and proper function in adipogenesis. Through tandem purification, we identified the molecular chaperone TRiC/CCT as WDTC1-interacting proteins. WDTC1 bound the TRiC/CCT through its ADP domain, and the TRiC/CCT recognized WDTC1 through the CCT5 subunit. Disruption of the TRiC/CCT by knocking down CCT1 or CCT5 led to misfolding and lysosomal degradation of WDTC1. Furthermore, the knockdown of CCT1 or CCT5 eliminated the inhibitory effect of WDTC1 on adipogenesis. Our studies uncovered a critical role of the TRiC/CCT in the folding of WDTC1 and expanded our knowledge on the regulation of adipogenesis.

## Introduction

WD40 and tetratricopeptide repeats 1 (WDTC1) is an anti-obesity factor conserved from worms to mammals (Suh et al., 2007). Mutation in *Adipose*, the *Drosophila* homolog of *WDTC1*, caused obesity in *Drosophila* (Doane, 1960a; Doane, 1960b; Hader et al., 2003). *Wdtc1* heterozygous mice are obese with enlarged adipocytes, whereas fat-specific *Wdtc1* transgenic mice are lean with dramatically decreased fat mass (Suh et al., 2007). In humans, the expression level of WDTC1 is negatively correlated with obesity (Lai et al., 2009; Galgani et al., 2013). WDTC1 is identified as an adaptor of the CRL4 E3 ligase complex (Groh et al., 2016). We have previously shown that the CRL4-WDTC1 complex targets MED20, a subunit of the mediator complex, for degradation to inhibit adipogenesis (Tang et al., 2021). MED20 directly binds C/EBPβ and is required for the transcription of C/EBPα and PPARγ, two central players in adipogenesis (Tang et al., 2021). However, it remains unclear about how WDTC1 is regulated.

The tailless complex protein-1 (TCP-1) ring complex (TRiC), also known as complex containing TCP-1 (CCT), is the primary eukaryotic cytosolic chaperonin that is essential for cytosolic protein folding and assembly (Lopez et al., 2015; Skjaerven et al., 2015). The TRiC/CCT consists of two stacked rings, which was composed of eight homologous but distinct subunits (CCT1-8) (Cong et al., 2010). The TRiC/CCT facilitates the folding of approximately 10% of the newly synthesized cytosolic polypeptides (Yam et al., 2008), including WD40 domain-containing proteins (Miyata et al., 2014; Lopez et al., 2015). Dysfunction of the TRiC/CCT has been associated with a number of diseases (Lopez et al., 2015), and its function is absolutely essential for viability (Yam et al., 2008). It remains unclear whether the TRiC/CCT regulates adipogenesis.

Here, in an effort to purify WDTC1-interacting proteins, we identified the TRiC/CCT as a major binding partner of WDTC1. The TRiC/CCT binds WDTC1 primarily through the CCT5 subunit and facilitates the folding of WDTC1. We demonstrate that the TRiC/CCT is required for the inhibitory effect of WDTC1 on adipogenesis. Our findings uncover a role of the TRiC/CCT in regulating adipogenesis.

## Materials and methods

We obtained Strep-Tactin Superflow (2-1206-002) and desthiobiotin (2-1000-001) from IBA life sciences; dexamethasone, isobutylmethylxanthine (IBMX), biotin, pioglitazone, bovine insulin, urea, SDS, DTT, DMSO, and Triton X-100 from Sigma-Aldrich; Dulbecco’s modified Eagle’s medium (DMEM) with low (1 g/L) or high (4.5 g/L) glucose, fetal and neonatal bovine serum, blasticidin, and puromycin from Thermo Fisher Scientific; donkey anti-rabbit IgG or anti-mouse IgG conjugated to horseradish peroxidase from Jackson Immuno Research; protease inhibitor cocktail from Roche Applied Science; and all other chemicals from local suppliers unless otherwise specified.

### Plasmids

Full-length cDNAs of human CCT1-8 subunits of the CCT were generous gifts from Dr. Jiahuai Han at Xiamen University, China. The coding regions of these genes were cloned into pcDNA3.3 (Thermo Fisher Scientific) with an N-terminal Flag tag. Mouse WDTC1 were cloned from a cDNA library prepared from testis mRNA of a C57BL6 mouse. A pIRESpuro-GLUE vector (Addgene, #15100) (Angers et al., 2006) was used to generate a stable cell line for the purification of WDTC1-interacting proteins. For lentiviral overexpression, genes were cloned into either pCDH-EF1-MCS-IRES-Puro (System Biosciences) or pCDH-EF1-MCS-IRES-BSD (modified from the former one by replacing the puromycin resistance gene to blasticidin resistant gene) with or without the N-terminal Flag tag. For knockdown of genes, a pLKO.1 vector (Addgene, 10878) (Moffat et al., 2006) was used. The primer sequences are listed in Supplementary Table S1.

### Culture of HEK293T cells and generation of stable cell lines

HEK293T cells were cultured as previously described (Wang et al., 2019; Hao et al., 2020). In brief, cells were cultured in Medium A (DMEM high glucose, 10% (v/v) FCS, 100 U/ml penicillin, and 100 mg/ml streptomycin) at 37°C in an atmosphere of 5% CO_2_. To generate stable cell lines, HEK293T cells were set up at 5×10^4^ cells per 10-cm dish on day 0. On day 1, cells were transfected with either 0.5 μg pIRESpuro-GLUE (pGlue) or WDTC1/pGlue using polyetherimide (PEI, Polysciences, 239660) at 2.4:1 (wt/wt) of PEI to plasmids. Starting from day 2, cells were selected with Medium A containing 1 μg/ml puromycin until colonies were formed, which were then screened by Western blot using the anti-HA antibody (1:2000, Roche, 11867423001). A positive clone of either construct was subcloned and used for the current studies.

### Culture and differentiation of 3T3-L1 cells

3T3-L1 preadipocytes were obtained from the American Type Culture Collection and cultured in Medium B (DMEM low glucose, 10% (v/v) NCS, 100 U/ml penicillin, and 100 mg/ml streptomycin) at 37°C in an atmosphere of 8.8% CO_2_. Cells were maintained at no more than 50% confluence.

To differentiate into mature adipocytes, 3T3-L1 cells were subjected to a standard cocktail hormone as previously described (Wang et al., 2021). In brief, cells were cultured to 100% confluence and maintained in Medium B for another 2 days. On day 0 of differentiation, cells were treated with Medium C (DMEM low glucose, 10% (v/v) FCS, 100 U/ml penicillin, and 100 mg/ml streptomycin) containing 5 μg/ml insulin, 0.5 mM IBMX, 1 μM dexamethasone, and 1 μM pioglitazone. On days 2 and 4, the medium was changed to Medium C containing 5 μg/ml insulin. On day 6, cells were fed with Medium C. On day 8, complete differentiation was achieved.

### Cell proliferation analysis

For the cell growth curve, control and CCT1/5 knockdown 3T3-L1 cells were set up at 1 × 10^4^ cells/35-mm dish. Cells were counted every day for 4 days.

### Oil Red O staining and quantification of triglycerides in 3T3-L1 cells

For Oil Red O staining, fully differentiated 3T3-L1 cells were washed with PBS and fixed in 2.5% glutaraldehyde at room temperature for 1 h. Cells were then treated with 60% isopropanol for 5 min and stained with freshly prepared Oil Red O (1.8 mg/ml) for 5 min. For quantification of triglycerides in 3T3-L1 adipocytes, cells were harvested in PBS containing 0.5% SDS, gradually heated up to 95°C, and kept at 95°C for another 5 min. The cooled samples were subjected to triglyceride measurement using a commercial kit from Wako Chemicals. The content of triglycerides was normalized to protein content, which was quantified by using a Pierce BCA kit (Thermo Fisher Scientific).

### Lentivirus production and infection

To produce lentivirus in HEK293T cells, on day 0, cells were set up at 2.5×10^5^ cells per 60-mm dish. On day 2, cells were transfected with 1 μg lentiviral vector and two packaging plasmids, 0.75 μg psPAX2 (Addgene, 12260) and 0.25 μg pMD2.G (Addgene, 12259), using polyetherimide (PEI, Polysciences, 239660). On day 3, cells were refed with Medium A. On days 4 and 5, lentiviral particles were collected from the media after centrifugation at 1,000 *g* for 5 min, concentrated at 70,000 *g* for 2 h or directly aliquoted, and stored at −80°C until further use.

For lentiviral infections, 3T3-L1 preadipocytes were set up, cultured to 50%–70% confluence, and infected with lentivirus in the medium containing 8–10 μg/ml polybrene. Cells, 24 h after infection, were selected against 5 μg/ml puromycin and/or 5 μg/ml blasticidin for at least 48 h and then used for the described experiments.

### Purification of WDTC1-interacting proteins

To purify WDTC1-interacting proteins, nuclear extracts were prepared from 10 of 10-cm dishes of stable cell lines of pGLUE or WDTC1/pGLUE as previously described (Nilsen, 2013). The nuclear extracts were incubated with 0.1 ml Strep-Tactin beads at 4°C for 1 h, washed with 10 ml Buffer A (10 mM HEPES, pH 8.0, and 150 mM NaCl), and eluted six times in 0.1 ml Buffer B (10 mM HEPES, pH 8.0, 150 mM NaCl, 10 mM β-mercaptoethanol, 1 mM MgOAc, 1 mM imidazole, 2 mM CaCl_2_, and 2.5 mM desthiobiotin). The eluted fractions were combined and incubated with 0.1 ml calmodulin beads at 4°C for 3 h, washed with 5 ml Buffer C (50 mM ammonium bicarbonate, pH 8.3, 75 mM NaCl, 1 mM MgOAc, 1 mM imidazole, and 2 mM CaCl2), and eluted in 0.1 ml Buffer B. The extracts were subjected to silver staining and protein ID analysis by AB5600+ mass spectrometry.

### Purification of recombinant WDTC1 and refolding of WDTC1

To express recombinant WDTC1, the coding region of WDTC1 was cloned into a pET28a vector and transformed into BL21(DE3)pLys bacterial cells. On day 0, a colony was inoculated in the LB medium and cultured at 37°C overnight. On day 1, the bacteria were scaled up at 1:100, cultured to an OD600 around 0.8, and induced with 1 mM IPTG for 3 h. Cells were then harvested and lysed in PBS containing 1 mg/ml lysozyme supplemented with protease inhibitors. As WDTC1 was expressed in the inclusion bodies, the pellet from the lysis was collected and thoroughly washed with PBS containing 1% Triton X-100 five times. The final product shows about 95% purity on SDS-PAGE.

To refold WDTC1 *in vitro*, purified WDTC1 proteins were denatured in 6 M urea in 10 mM HEPES pH 8.0 at 25°C overnight. On the next day, cytosolic fractions were prepared from cells infected with control shRNA, shCC1, or shCCT5. In brief, cells were harvested by spinning at 1,000 *g* for 5 min and resuspended in hypotonic buffer (10 mM HEPES, pH 7.9, 1.5 mM MgCl_2_, and 10 mM KCl) to swell for 10 min on ice. Cells were homogenized in a Dounce homogenizer with 10 strokes using a loose-fitting pestle and spun at 1,000 *g* for 15 min. The resultant was supplemented with 0.11 volume of 10× cytoplasmic extract buffer (0.3 M HEPES, pH 7.9, 1.4 M KCl, and 0.03 M MgCl_2_) and spun at 100,000 *g* at 4°C for 1 h. The supernatant is the cytosolic fraction. The denatured WDTC1 proteins (100 ng) were diluted into cytosolic fractions and incubated at 25°C for 1 h. The samples were then centrifuged at 20,000 *g* for 10 min, and the supernatant and pellet fractions were subjected to SDS-PAGE and Western blot.

### Western blot

Western blot was carried out as previously described (Zhang et al., 2015). The following antibodies were used: anti-CCT1 (1:1,000, Protein Tech, 10320-1-AP), anti-CCT5 (1:1,000, Protein Tech, 11603-1-AP), anti-WDTC1 (1:1,000, Abgent, AP4944b), anti-CD36 (1:1,000, Sino Biological Inc., 80263-T48), anti-PPARγ (1:1,000, Santa Cruz, sc-7273), anti-C/EBPα (1:1,000, CST, 8178s), anti-perilipin (1;1,000, CST, 9349s), anti-GAPDH (1:5,000, CST, 5174s), and anti-Flag M2 (1:1,000, Sigma, F1804). Membranes were developed in a ChemStudio imaging system (Analytik Jena AG).

### Quantification and statistical analysis

All the statistical analyses were performed using Student’s two-tailed paired t-test. The value represents mean ± SEM. Statistical details of all experiments can be found in the figure legends, including the exact number of cell samples. Asterisks (*) indicate levels of statistical significance. *, *p* < 0.05; **, *p* < 0.01; and ***, *p* < 0.001. No data were excluded from any of the experiments.

## Results

### Identification of the CCT as WDTC1-interacting proteins

**FIGURE 1:**
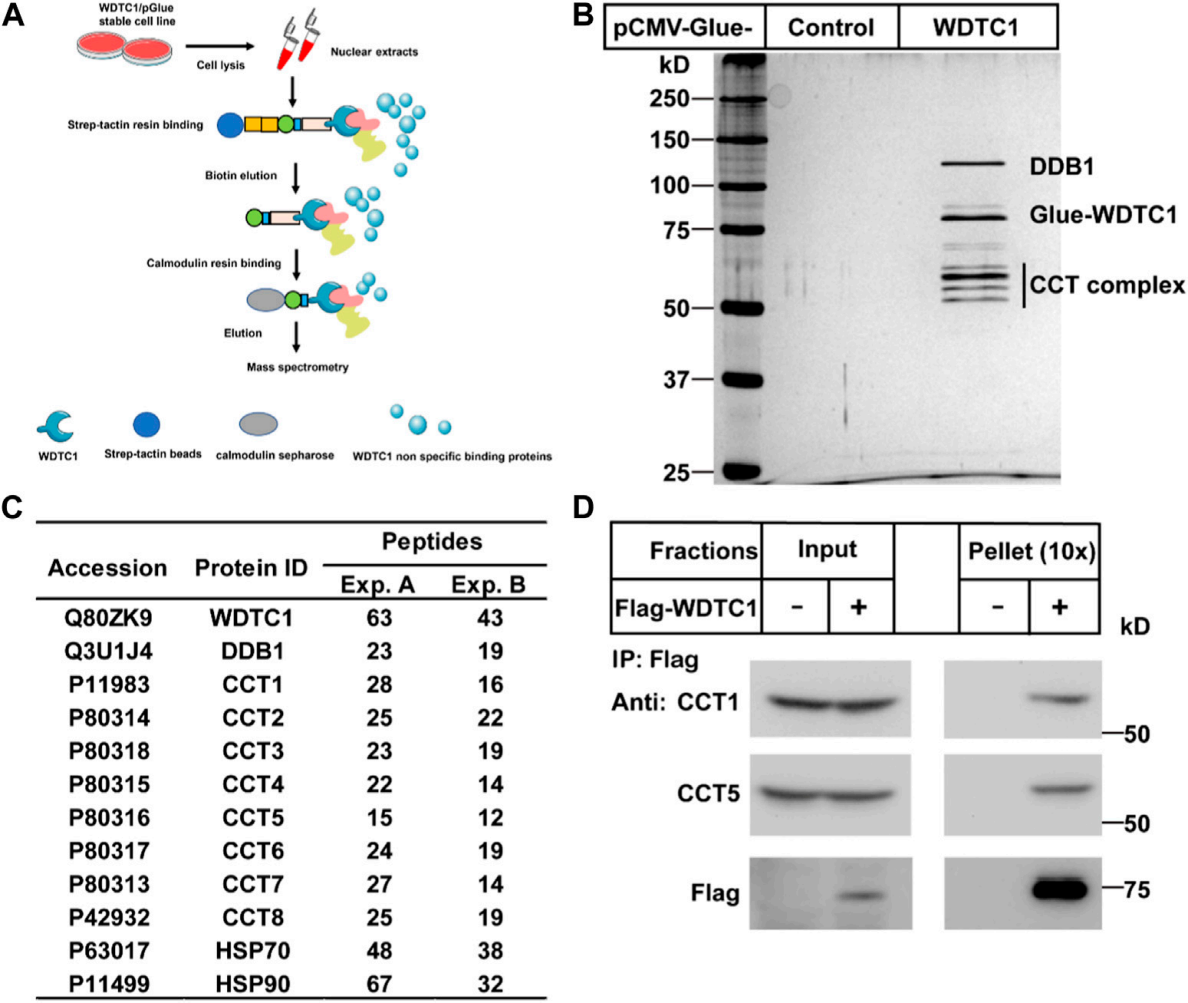
Identification of the CCT complex as WDTC1-interacting proteins. **(A)** Strategy to purify WDTC1-interacting proteins in HEK293T cells using Glue-WDTC1 as a bait. **(B,C)** On day 0, nuclear fractions were prepared from stable HEK293T cells expressing WDTC1/pGlue or vector alone and subjected to purification with Strep-Tactin beads as described. The eluted fractions were subjected to silver staining (B) and mass spectrometry for protein ID analysis. Potential candidates are listed in (C). **(D)** On day 0, HEK293T cells were set up at 250,000 cells/60-mm dish. On day 2, cells were transfected with pEF-Flag-WDTC1 or vector alone. On day 3, immunoprecipitation was performed using anti-Flag M2 beads and endogenous CCT1 and CCT5 were detected.

To search for potential interacting proteins of WDTC1, we generated a stable HEK293T cell line expressing Glue (SBP-HA-CBP)-tagged WDTC1 and performed tandem purification (Figure 1A). When subjected to silver staining, several bands were specifically identified in the Glue-WDTC1-expressing cells (Figure 1B). Mass spectrometry analysis showed that these bands contained DDB1, all eight subunits of the TRiC/CCT, Hsp70, and Hsp90, which was confirmed by two separate experiments (Figure 1C).

The chaperonin TRiC/CCT has been shown to facilitate the folding of WD40-domain containing proteins (Miyata et al., 2014; Lopez et al., 2015). As WDTC1 is also a WD40-domain containing protein, we hypothesized that the TRiC/CCT might be required for the folding and function of WDTC1. We first overexpressed Flag-tagged WDTC1 in HEK293T cells and performed co-immunoprecipitation (co-IP) assay. As shown in Figure 1D, WDTC1 pulled down endogenous CCT1 and CCT5, indicating that the TRiC/CCT indeed binds WDTC1.

### WDTC1 binds the TRiC/CCT through its ADP domain

**FIGURE 2:**
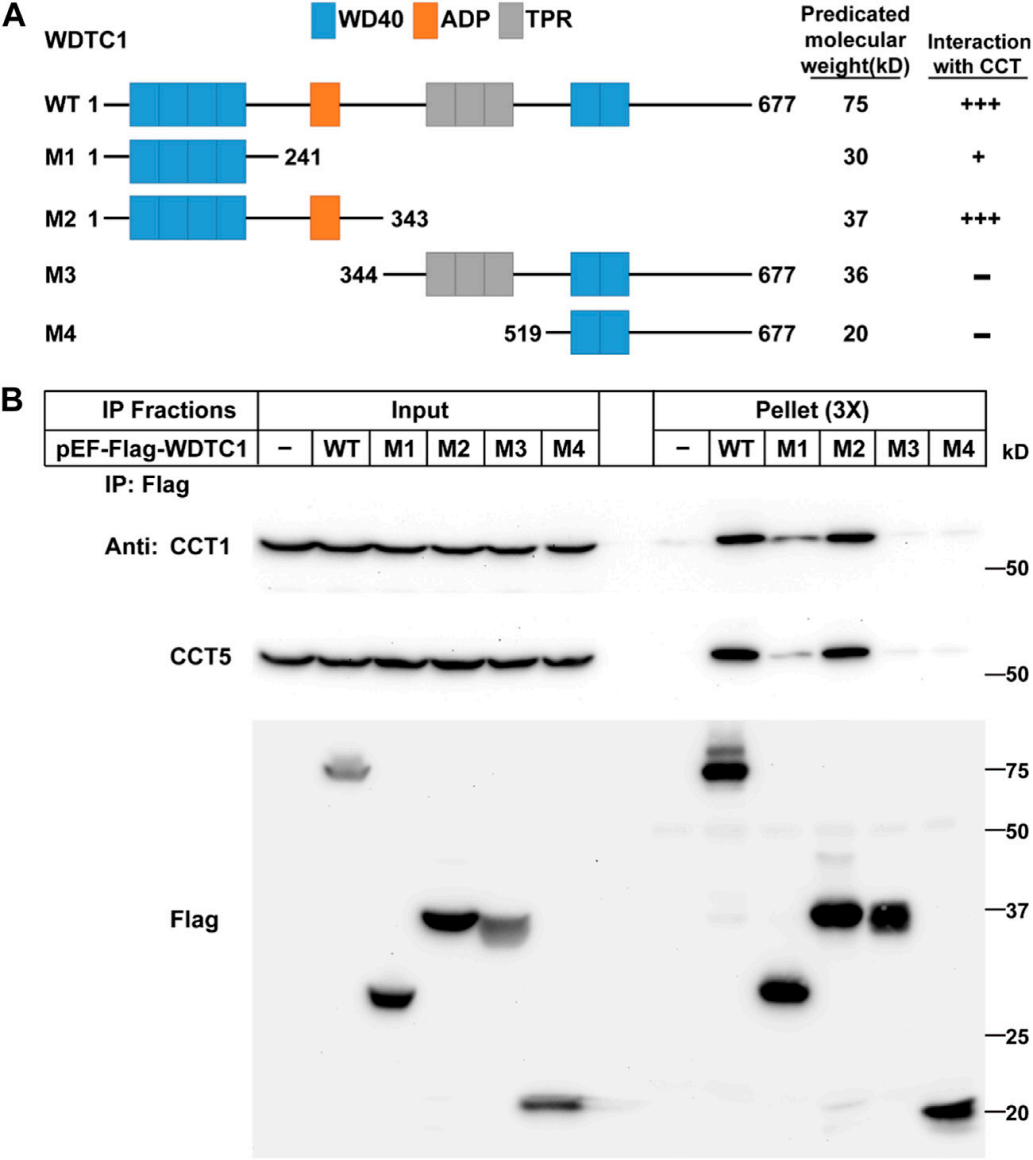
WDTC1 interacts with the CCT through its ADP domain. **(A)** Illustration of the domain structures of WT and different truncates of WDTC1. **(B)** HEK293T cells were set up, transfected with indicated plasmids, and subjected to immunoprecipitation as in Figure 1D. Input and pellet fractions were immunoblotted with indicated antibodies.

We next examined which part of WDTC1 binds the TRiC/CCT. Previous studies have shown that WDTC1 has six WD40 domains, one ADP domain, and three tetratricopeptide domains (TPR) (Hader et al., 2003). To map the interaction domain of WDTC1 with the CCT, we generated different truncates of WDTC1 (Figure 2A) and transfected them into HEK293T cells for co-IP assay. As shown in Figure 2B, the M1 truncate (aa1-241) of WDTC1 pulled down a small fraction of endogenous CCT1 and CCT5, but M2 truncate (aa 1–343) pulled down comparable amounts of CCT1 and CCT5 with full-length WDTC1, suggesting that the ADP domain is the primary domain that interacts with the TRiC/CCT. Consistently, the M3 and M4 truncates, which lacked the ADP domain, showed no interaction with the TRiC/CCT.

### TRiC/CCT binds WDTC1 through CCT5

**FIGURE 3:**
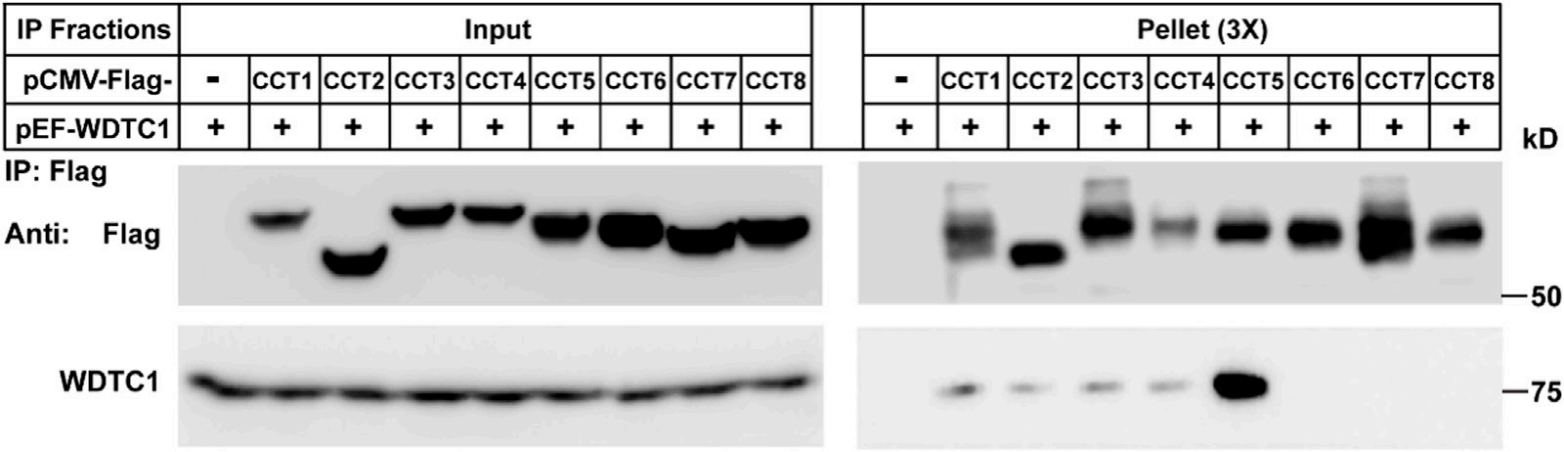
TRiC/CCT binds WDTC1 through CCT5. On day 0, HEK293T cells were set up at 250,000 cells/60-mm dish. On day 2, cells were transfected with pEF-Flag-CCT1-8 or vector alone. On day 3, immunoprecipitation was performed using anti-Flag M2 beads and endogenous WDTC1 were detected.

We then determined which subunit of the TRiC/CCT interacts with WDTC1. We co-transfected WDTC1 with each of the Flag-tagged subunit of the TRiC/CCT into HEK293T cells and performed a Co-IP experiment. As shown in Figure 3, the immunoprecipitation of Flag-CCT5 pulled down comparable amounts of WDTC1 than other subunits, indicating that the TRiC/CCT binds WDTC1 primarily through CCT5.

### TRiC/CCT is required for the stability of WDTC1

**FIGURE 4:**
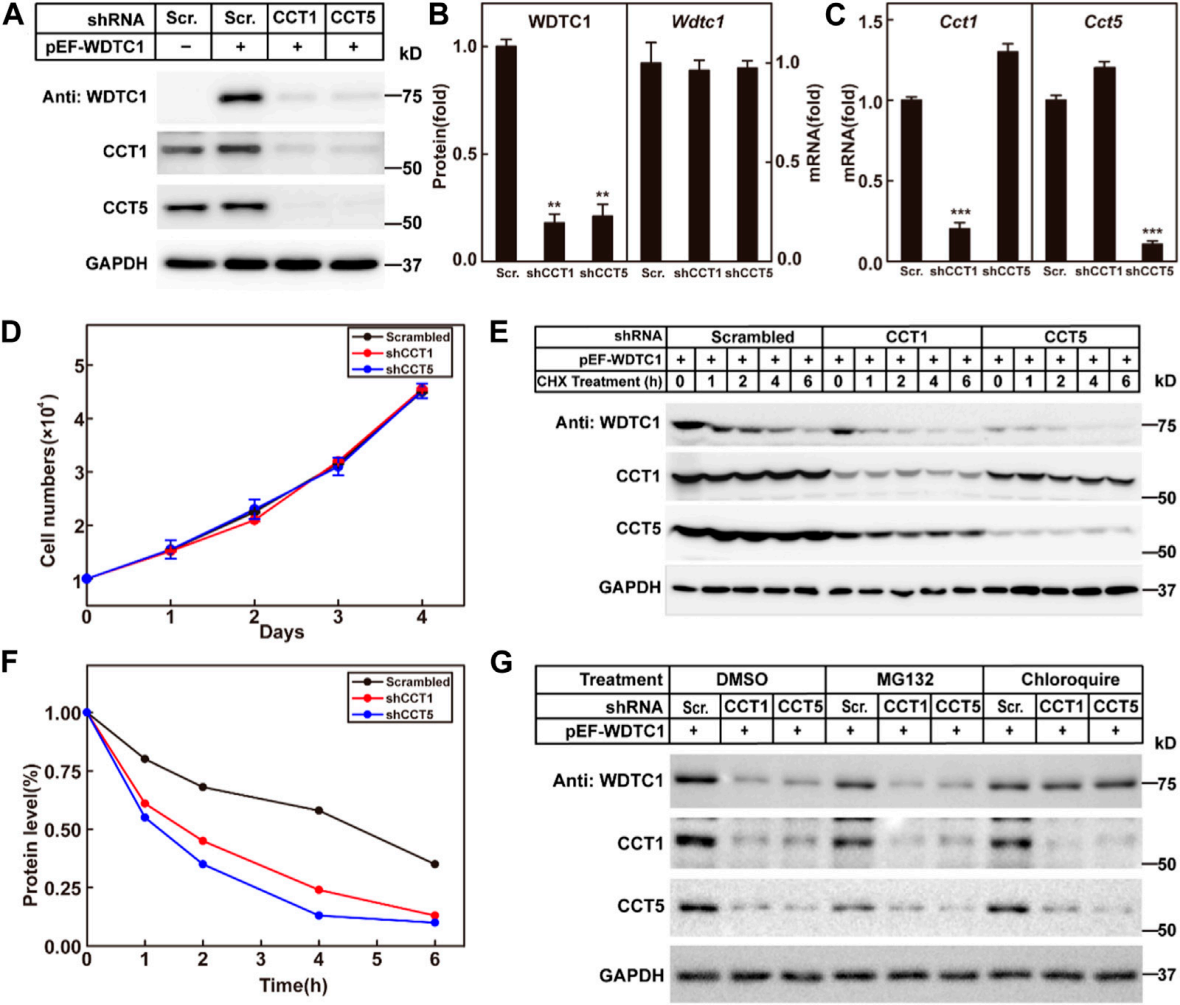
TRiC/CCT is required for the stability of WDTC1. **(A–C)** On day 0, 3T3-L1 preadipocytes infected with lentivirus expressing WDTC1 and the indicated shRNAs were set up at 1 × 10^5^ cells per 35-mm dish. On day 2, cells were harvested to detect the protein (A) and RNA (B, C) levels of WDTC1, CCT1, and CCT5. Each value represents mean ± SEM of three replicates. **(D)** Control, CCT1, and CCT5 knockdown 3T3-L1 cells were set up at 1 × 10^4^ cells/35-mm dish. Cells were counted every day for 4 days. Cell numbers are plotted in (D). **(E,F)** On day 0, 3T3-L1 preadipocytes infected with lentivirus expressing the indicated shRNAs and WDTC1 were set up at 1 × 10^5^ cells per 35-mm dish. On day 2, cells were treated with CHX (100 μg/ml) for 0, 1, 2, 4, and 6 h, then cells were harvested, and total cell lysate was immunoblotted with indicated antibodies (E); band intensities of WDTC1 are quantified and plotted in (F). **(G)** On day 0, 3T3-L1 preadipocytes infected with lentivirus expressing WDTC1 and the indicated shRNAs were set up at 1 × 10^5^ cells per 35-mm dish. On day 2, cells were treated with MG132(10 μg/ml) or chloroquine (50 μM) for 6 h, then cells were harvested, and total cell lysate was immunoblotted with indicated antibodies. For all panels, asterisks (*) denote the level of statistical significance (Student’s t-test). **p < 0.01 and ***p < 0.001.

We then went on to check the effect of the TRiC/CCT on WDTC1. We generated a stable cell line in 3T3-L1 cells that overexpress WDTC1 and knocked down CCT1 or CCT5 to check their effect on the protein and RNA levels of WDTC1. Consistent with the work of Trinidad et al. (2013), knockdown of either CCT1 or CCT5 resulted in a decreased protein level of the other (Figures 4A, C), suggesting that all subunits are required for the integrity of the TRiC/CCT. Depletion of either CCT1 or CCT5 dramatically reduced the protein, but not the mRNA, level of WDTC1 (Figures 4A–C), indicating that the TRiC/CCT is required for the stability of WDTC1. Moreover, knockdown of CCT1 or CCT5 showed no difference on cell viability with control (Figure 4D).

To further confirm the results, we overexpressed WDTC1 in control and CCT1 or CCT5 knockdown 3T3-L1 cells, treated with cycloheximide, and examined the half-life of WDTC1. As shown in Figures 4E, F, the half-life of WDTC1 in the control cells was about 4 h, but it was shortened to about 2 h in CCT1 or CCT5 knockdown cells.

To examine how WDTC1 was degraded in cells lack of the TRiC/CCT, we treated CCT1 or CCT5 knockdown 3T3-L1 cells with MG132 or chloroquine and found that chloroquine, but not MG132, largely restored the level of WDTC1, suggesting a lysosomal degradation of WDTC1 in TRiC/CCT-depleted cells (Figure 4G).

### TRiC/CCT is required for the protein folding of WDTC1

**FIGURE 5:**
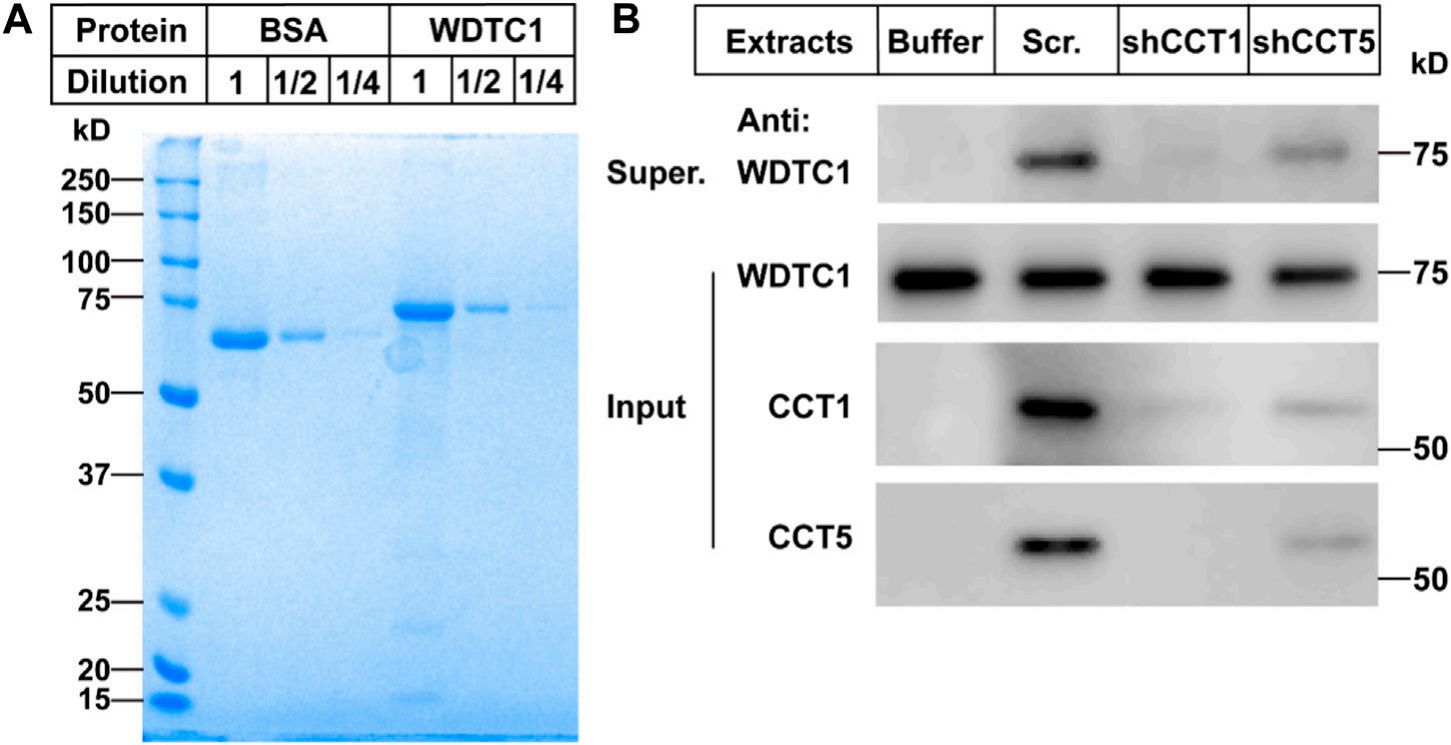
TRiC/CCT is required for the protein folding of WDTC1. **(A)** On day 0, the WDTC1 colony was incubated in the LB medium overnight. On day 1, the bacteria were induced with 1 mM IPTG for 3 h. Cells were then harvested and lysed in PBS containing 1 mg/ml lysozyme supplemented with protease inhibitors. The final product was subjected to SDS-PAGE and Coomassie Blue Staining. **(B)** On day 0, the purified WDTC1 was then denatured in 6 M urea in 10 mM HEPES pH 8.0. On day 1, 100 ng denatured WDTC1 protein was diluted into cytosolic fractions prepared from 3T3-L1 cell medium buffer or cells infected with scrambled shRNA, shCC1, or shCCT5 and incubated at 25°C for 1 h. The supernatant and pellet fractions of samples were immunoblotted with indicated antibodies.

To directly test whether the TRiC/CCT was required for the folding of WDTC1, we carried out an *in vitro* refolding assay. We expressed WDTC1 in *E. coli* and purified it from the inclusion bodies (Figure 5A). The purified WDTC1 was then denatured in urea and diluted to allow refolding in the cytosol prepared from control (scrambled shRNA), CCT1, or CCT5 knockdown 3T3-L1 cells. As shown in Figure 5B, WDTC1 was undetectable in the supernatant when refolded in buffer alone, whereas a significant amount of WDTC1 was detected in the cytosol prepared from the control cells. However, in the cytosol of either CCT1 or CCT5 knockdown cells, the amount of WDTC1 in the supernatant was almost undetectable, indicating that the TRiC/CCT was required for the refolding of WDTC1.

### TRiC/CCT is required for the inhibitory effect of WDTC1 on adipogenesis

**FIGURE 6:**
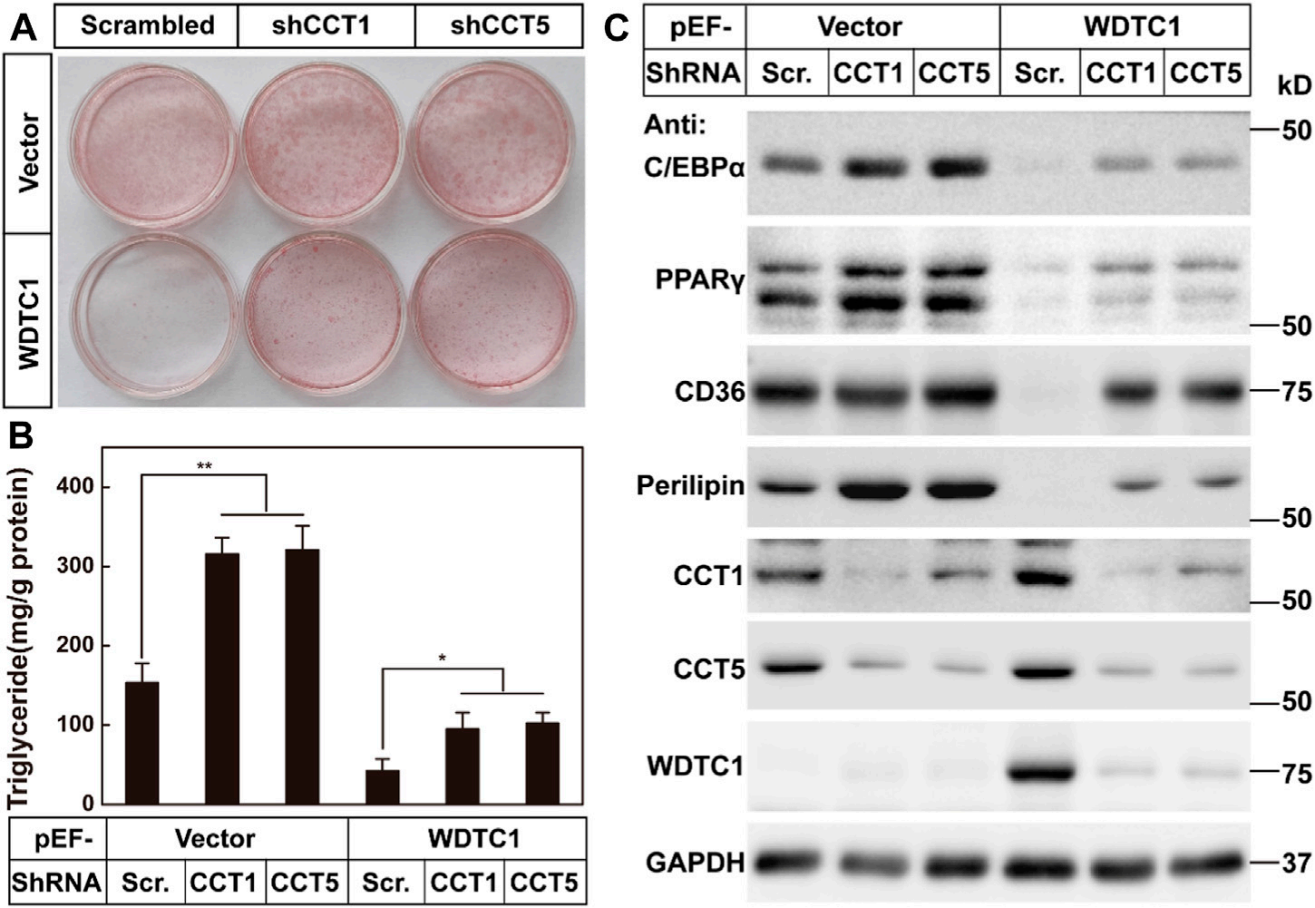
TRiC/CCT is required for the inhibitory effect of WDTC1 on adipogenesis. **(A–C)** 3T3-L1 preadipocytes infected with lentivirus expressing WDTC1 and the indicated shRNAs on pLKO.1 vector were set up at 1 × 10^5^ cells per 35-mm dish and subjected to differentiation. On day 8 of differentiation, cells were harvested for Oil Red O staining (A), measurement of intracellular triglyceride content (B), and Western blot tests (C). Each value represents means ± SEM of three samples. Asterisks (*) denote the level of statistical significance (Student’s t-test) between scrambled and shCCT1 or shCCT5 cells; *p < 0.05 and **p < 0.01.

We then went on to test the effect of the TRiC/CCT on the inhibitory effect of WDTC1 on adipogenesis. We overexpressed WDTC1 in 3T3-L1 cells, knocked down either CCT1 or CCT5, and then, subjected the cells to differentiation. After 8 days of differentiation, while the overexpression of WDTC1 suppressed differentiation, knockdown of either CCT1 or CCT5 largely restored the differentiation, as illustrated by lighter Oil Red O staining, intracellular triglyceride content, and the expression of the adipocyte markers (Figures 6A–C). These results indicate that the TRiC/CCT is required for the inhibitory effect of WDTC1.

## Discussion

Adipogenesis is closely associated with obesity and related metabolic disorders (Rosen and MacDougald, 2006). Over the decades, numerous factors have been identified as key regulators of adipogenesis (Siersbaek et al., 2012). Among them, WDTC1 is very intriguing, as it is an evolutionally conserved anti-obesity factor (Hader et al., 2003; Suh et al., 2007). Functioning as an adaptor of the CRL4 E3 complex, WDTC1 binds MED20, which is essential for the transcriptional activation of C/EBPβ, and leads to its ubiquitination and degradation to inhibit adipogenesis (Tang et al., 2021). Here, we identify the TRiC/CCT as a key regulator of WDTC1. The TRiC/CCT binds WDTC1 and facilitates its protein folding and function. Disruption of the complex leads to the degradation of WDTC1 and eliminates the inhibitory effect of WDTC1 on adipogenesis. Our findings help better understand the regulatory mechanism of WDTC1 and adipogenesis.

In addition, we demonstrated a clear role of the TRiC/CCT in regulating adipogenesis. The TRiC/CCT is a common molecular chaperone found in all eukaryotes, and it is involved in many cellular activities by helping the folding of its substrates (Sternlicht et al., 1993). WD40-containing proteins have been shown to be substrates of the TRiC/CCT. Here, we clearly proved that WDTC1 is a substrate of the TRiC/CCT and, thus, expanded the role of the complex in adipogenesis. Furthermore, knockdown of either CCT1 or CCT5 largely promotes differentiation and lipid accumulation, suggesting that the endogenous WDTC1 might be regulated by the TRiC/CCT. However, we cannot rule out whether there are other substrates of the TRiC/CCT that might also be involved in adipogenesis. In the future, it will be interesting to find whether there are clinical mutants of the TRiC/CCT that are closely associated with obesity and related metabolic diseases.

Moreover, there are also some limitations of this study. In the adipogenesis study, we only choose 3T3-L1 preadipocytes, which, although widely applied in adipogenesis (Kuri-Harcuch et al., 2019), cannot really reflect human adipogenesis. Some other types of adipocytes need to be tested in the future study. Additionally, the roles of WDTC1 and the TRiC/CCT in other cell types also remain elusive.

## Data Availability

All data generated or analyzed during this study are included in this published article and its Supplementary Information Files.
