# Supplementary material for: TRiC/CCT chaperonin is required for the folding and inhibitory effect of WDTC1 on adipogenesis
Source: Front Cell Dev Biol. 2023 Aug 24;11:1225628. doi: 10.3389/fcell.2023.1225628 (PMC10483223; doi:10.3389/fcell.2023.1225628)
Supplement: Supplementary file 1 [file Table1.DOCX]

**Supplemental Table S1**

| Primers | Source of Primer sequences |
| --- | --- |
| **A. Primers to generate different constructs** | |
| mCCT1-shRNA1 | CCTAATACACTGGCAGTGAAT |
| mCCT1-shRNA2 | CCTGAGAAATTGGACCAAATT |
| mCCT5-shRNA1 | GCTGGGCTCCAAAGTGATTAA |
| mCCT5-shRNA2 | GCCACCTAAACCTAAGACAAA |
| mWDTC1-F- | ATGGCAAAAGTCAACATAAC |
| mWDTC1-R- | CTAGCTGGGCCGGCACTGCAC |
| Flag-mWDTC1-F | ATGGACTACAAAGACGATGACGACAAGGCAAAAGTCAACATAACTAGAG |
| M1-241-R | TCAGGGCTTCTGTCGGTCACAG |
| M2-343-R | CTACCGGAAGCCGTTGCTGTGAAG |
| M3-344-F | ATGGAACAAAAACTCATCTCAGAAGAGGATCTGCCAGAGAGCAAGGGGTG |
| M4-519-F | ATGGAACAAAAACTCATCTCAGAAGAGGACCTACGGGAACGGAGCTACG |
| hCCT1-F | GAGGGGCCTTTGTCCGTGTTC |
| hCCT1-R | TCAATCATTAAGGGCTCCAG |
| hCCT2-F | GCGTCCCTTTCCCTTGCACC |
| hCCT2-R | TTAACAGGGGTGGTGATCAGG |
| hCCT3-F | GGCCATCGTCCAGTGCTCG |
| hCCT3-R | TCACTCCTGGCCAGCATCAG |
| hCCT4-F | CCCGAGAATGTGGCACCCC |
| hCCT4-R | TTATCGAGTGTTTACCACATC |
| hCCT5-F | GCGTCCATGGGGACCCTCGC |
| hCCT5-R | TCATTCTTCAGATTCTCCAG |
| hCCT6-F | GCGGCGGTGAAGACCCTGAAC |
| hCCT6-R | TCAACCTTTCAGAGAAGACA |
| hCCT7-F | CCCACACCAGTTATCCTATTG |
| hCCT7-R | TCAGTGGGGGCGGCCACGAC |
| hCCT8-F | GCGCTTCACGTTCCCAAGGC |
| hCCT8-R | TCAATCATTTTGGTCATCATCCC |
| **B. Quantitative real-time PCR Primers** | |
| mWDTC1 | GATGGGCTTATCCGCCAGTAT  GGTTGACGGTGAGGCACTT |
| mCCT1 | CCGCTCCCAGAATGTTATGG  CGGGATGTTCTACCTCCAGT |
| mCCT5 | ACCCTCGCCTTCGATGAGTAT  GCATTGTGTTTGCTACAGCTTT |
